# Fecal bacterial biomarkers and blood biochemical indicators as potential key factors in the development of colorectal cancer

**DOI:** 10.1128/msystems.00043-25

**Published:** 2025-02-27

**Authors:** Ping Cai, Qingzhen Yang, Jiaqi Lu, Xiaoyu Dai, Jinbo Xiong

**Affiliations:** 1 Ningbo No.2 Hospital, Ningbo, China; 2 The Key Laboratory of Biomedical Information Engineering of Ministry of Education, School of Life Science and Technology, Xi’an Jiaotong University, Xi’an, China; 3 Bioinspired Engineering and Biomechanics Center (BEBC), Xi’an Jiaotong University, Xi’an, China; 4 Zhejiang KinGene Bio-technology Co., Ltd, Ningbo, China; 5 Institute of One Health, School of Marine Sciences, Ningbo University, Ningbo, China; Argonne National Laboratory, Lemont, Illinois, USA

**Keywords:** colorectal cancer, polyps, random forest model, fecal microbiome, blood biochemical indicators

## Abstract

The incidence of colorectal cancer (CRC) has been increasing in recent decades. Current methods for CRC screening have their own drawbacks, thus there is an urgent need to identify the key microbes that drive the development of CRC for wider application in the early detection and prevention of CRC. To address this issue, we performed fecal microbiome analysis by high-throughput sequencing of 16S rRNA gene combined with blood biochemical indicators in patients with CRC stages I, II, III, and IV, healthy people, and patients with polyps. Fecal microbiota of patients with CRC was disturbed, as evidenced by significantly reduced α-diversity in patients with CRC stage IV and markedly different β-diversity. The random forest model identified the top 25 genera from 174 training data, resulting in a diagnostic accuracy of 87.95%. Further, by combining with differential genera analysis, we screened out 11 biomarkers that significantly changed in different groups. *Peptostreptococcus*, *Parvimonas*, *Shewanella*, *Oscillibacter*, *Eggerthella*, and *Gemella* associated with the development of CRC were significantly enriched, while *Fenollaria*, *Staphylococcus*, *Ezakiella*, *Finegoldia,* and *Neisseria* associated with the remission of CRC were significantly suppressed in patients with CRC. Importantly, carcinoembryonic antigen (CEA) was significantly correlated with these 11 microbial biomarkers, and carbohydrate antigen 19-9 (CA 19-9) was markedly correlated with *Oscillibacter*. Notably, co-occurrence network analysis at the genus level exhibited that the microbial co-occurrence network of CRC IV was the most complex and stable. These results suggested that CEA, CA 19-9 and 11 microbial biomarkers may be co-biomarkers for the disease occurrence and development, and non-invasive diagnosis of CRC.

**IMPORTANCE:**

Identifying the key microbes that drive the development of colorectal cancer (CRC) has been important in this field. We delved into the research on the association between CRC and fecal microbiota in this study, providing a detailed analysis of the characteristics of fecal microbiota during the transition from normal intestine to polyps to cancer. Fecal bacterial biomarkers and blood biochemical indicators may be co-biomarkers in the development of CRC.

## INTRODUCTION

Colorectal cancer (CRC) is a common malignant tumor caused by malignant transformation of intestinal polyps (1). CRC is the most common and deadliest cancer in the world except lung cancer, and 10% of new cancers are CRC (2). Globally, Western countries have the highest CRC morbidity and mortality rates (3). However, the data on CRC morbidity and mortality rates are not optimistic for China. As of 2022, CRC ranks high among all cancers in terms of incidence and mortality (4). Early diagnosis of CRC can significantly improve the cure rate (5). Therefore, early identification of colorectal polyps and precancerous lesions, along with in-depth research into their influencing factors, is crucial for reducing the incidence of CRC.

Early detection of CRC is crucial, which can raise the patients' 5-year survival rate to about 90%. Therefore, timely identification of CRC has profound significance for extending the patient’s survival period (6). Currently, the main methods for CRC screening are imaging and stool-based tests (7). Colonoscopy is a key method for diagnosing CRC, but the procedure may be associated with complications such as bleeding and perforation (8, 9). Furthermore, endoscopic examination may increase the patients' financial burden and physical risks, leading to low compliance with the examination (10, 11). This low compliance may hinder the early detection of CRC, thereby affecting the patient’s survival rate and treatment outcomes. Stool-based test methods, such as fecal immunochemical tests (FITs) and fecal occult blood tests (FOBTs), offer a convenient and relatively low-cost approach to CRC screening (12). However, FIT is relatively expensive, and its sensitivity needs to be improved. The sensitivity and specificity of FOBT are insufficient, making it prone to false positives due to other factors, which may cause unnecessary anxiety and additional medical examinations (13). Overall, although colonoscopy has the advantage of accuracy, it is not suitable as a routine screening method for all patient groups due to resource limitations and patient acceptance issues. Therefore, there is an urgent need to develop an efficient and safe screening method that can be more widely applied in the early detection and prevention of CRC.

The high incidence of CRC may be related to environmental changes. Among environmental factors, gut microbiota is involved in the occurrence and development of tumors (14). The gut microbiome is a complex community of various symbiotic and non-symbiotic microorganisms that reside in the intestines (15–17). The tumor microenvironment of CRC is a complex ecosystem composed of mutated cancer cells, a variety of non-tumor cells, and microbial communities. Each component may contribute to tumor initiation and progression (18). The gut microbiota plays an important role in the pathogenesis of CRC through microbes and their metabolic products (19). The previous study found that *Fusobacterium* DNA was enriched in colorectal tumors relative to normal samples using fuorescence *in situ* hybridization. For the first time, colorectal tumor specimens were associated with genus *Fusobacteria* (20). In past studies, a growing number of studies have demonstrated a strong link between CRC and microbiota (21, 22). CRC-associated enriched strains, such as *Parvimonas micra*, *Porphyromonas asaccharolytica*, *Prevotella intermedia,* and *Alistipes finegoldii*, were further identified by culture and quantitative real-time polymerase chain reaction (qPCR) (23–25). The importance of the gut microbiome in cancer screening cannot be overstated, as it has the potential to usher in a new era of cancer screening. The prediction of CRC by mapping intestinal microbial characteristics from fecal samples may have a positive impact on the prevention, screening, and treatment of CRC (26). With the scientific community gradually recognizing the key role of the gut microbiome in the tumor microenvironment, it is urgent to explore a comprehensive screening method that combines the gut microbiome with traditional screening methods.

Liquid biopsy technology has been increasingly refined in the field of cancer management. By detecting specific biomarkers in plasma, it offers tremendous potential for precision medicine. Research and application in this field have garnered widespread attention in recent years, with the expectation of further enhancing the accuracy of cancer diagnosis and treatment (27). In plasma, three novel methylated DNA biomarkers have successfully identified 86% of gastric adenocarcinoma cases from the normal control group with a specificity of up to 95% (28). Furthermore, six serum microRNAs (miRNAs) have been utilized to differentiate between gastric cancer patients and healthy controls, demonstrating their potential in cancer diagnostics (29). Over the past few decades, researchers have introduced serum tumor markers for breast cancer screening (30). Blood biochemical indicators are widely applied due to their convenience, non-invasive nature, and effectiveness. However, the differences in the stability of biomarkers among individuals may affect the accuracy of liquid biopsies, limiting their application in clinical practice (31). Therefore, integrating gut microbiome data with the analysis of blood biochemistry can significantly enhance the reliability of detection, making disease screening more accurate and efficient.

Identifying the key microbes that drive the development of CRC has always been at the heart of research in this field. We delved into the research on the association between CRC and fecal microbiota in this study, providing a detailed analysis of the characteristics of fecal microbiota during the transition from normal intestine to polyps to cancer. We identified key fecal microbes that are crucial for screening polyps or CRC. Furthermore, through in-depth analysis of the correlations between blood biochemical indicators and fecal microbiota, we identified key fecal microorganisms and blood indicators, which provide a solid scientific basis for understanding the mechanism of cancer occurrence and development and formulating effective prevention and treatment strategies.

## MATERIALS AND METHODS

### Subjects

The subjects were those who underwent standard colonoscopy at Ningbo No.2 Hospital. This study included 479 samples collected over a 5-year period from Ningbo No.2 Hospital. Exclusion criteria included anal canal tumors, appendiceal tumors, neuroendocrine tumors, familial adenomatous polyposis, patients who underwent additional surgery after endoscopic treatment with perforation or bleeding, patients who used antibiotics, glucocorticoids, or immunosuppressants within 1 month of biopsy, and other patients with CRC who could not enter the cohort (32). Patients were staged according to histopathological features of the Tumor, Nodes, and Metastasis (TNM) staging system. Samples with indeterminate TNM staging were excluded from the analysis if there was insufficient clinical evidence (33). All volunteers were divided into six groups: C1 (35 samples), C2 (101 samples), C3 (110 samples), and C4 (15 samples) groups corresponded to CRC stages I, II, III, and IV, respectively. The H group (120 samples) were healthy volunteers, and the P group (92 samples) were patients with polyps. For all volunteers, approximately 5 g fresh feces was collected from the midsection as samples, immediately frozen at −80°C and transported to the laboratory. Blood and feces samples were collected from volunteers and stored at −80°C until further processing. The studies involving human participants were reviewed and approved by the ethics committees of Ningbo No.2 Hospital (No. PJ-NBEY-KY-2020-042-01). The participants provided their written informed consent to participate in this study.

### Serum clinical biochemistry

Blood samples were gathered to quantitatively measure various biochemical indicators in the serum using the Ortho VITROS 5600 blood biochemical analyzer (34), which included carcinoembryonic antigen (CEA), carbohydrate antigen 19-9 (CA 19-9), cholesterol, triglycerides, blood glucose, and carbohydrate antigen 125 (CA 125).

### High-throughput sequencing of fecal microbiota

Fecal microbiota was analyzed using the high-throughput sequencing of 16S rRNA gene. We extracted the total DNA using feces using the DNA extraction kit (Biogoethe Biotechnology Co., Ltd., Wuhan, China). Following Illumina’s protocol for 16S metagenomic library preparation, we created a sequence library of the 16S rRNA gene amplicons. These were then pooled in equimolar amounts and subjected to paired-end sequencing (2 × 300 bp) on an Illumina MinSeq platform by Shanghai Majorbio Bio-pharm Technology Co., Ltd. Post-sequencing, sequences were demultiplexed and merged using FLASH (version 1.2.11), followed by quality filtering with fastp (version 0.19.6). High-quality sequences were further processed with the Deblur plugin in Qiime2 (2020.2 version) for de-noising, resulting in amplicon sequence variants (ASVs) (35).

### Statistical analysis

The Vegan package based on R language was used to analyze the α-diversity, β-diversity, and heatmap at the genus level of fecal microorganisms. α-diversity was used to analyze the number of species and evenness of fecal microbial communities in the samples. β-diversity, including calculation of the Bray-Curtis matrix and principal coordinate analysis of the constrained principal coordinate analysis (CPCoA), were used to visualize and analyze the non-similarity of fecal microbial community composition among treatments. To pinpoint gut bacterial biomarkers of subjects' health conditions, we developed a diagnostic model employing the random forest (RF) method. This was done post-evaluation of how patients with CRC stages I, II, III, and IV, healthy people, and patients with polyps uniquely influence the alterations in gut microbiota (36). Similarity analysis (ANOSIM) was further used to quantitatively assess their relative importance in controlling the gut microbiota of the subjects using the “Adonis” function (37). Spearman correlation analysis of the bacterial genera was carried out using R software, with *P* values < 0.05 considered statistically significant. Co-occurrence network analysis visualization was conducted using Gephi 0.10.1 software. Statistical analysis and plotting were performed using GraphPad software (8.0 version, USA) and R Programming Language (R 4.1.0, New Zealand). A two-tailed Student’s *t* test or one-way analysis of variance (ANOVA) was conducted to interpret significant differences among different groups, with *P* < 0.05 considered significant.

## RESULTS

### Comparison of fecal microbial diversity

**Fig 1:**
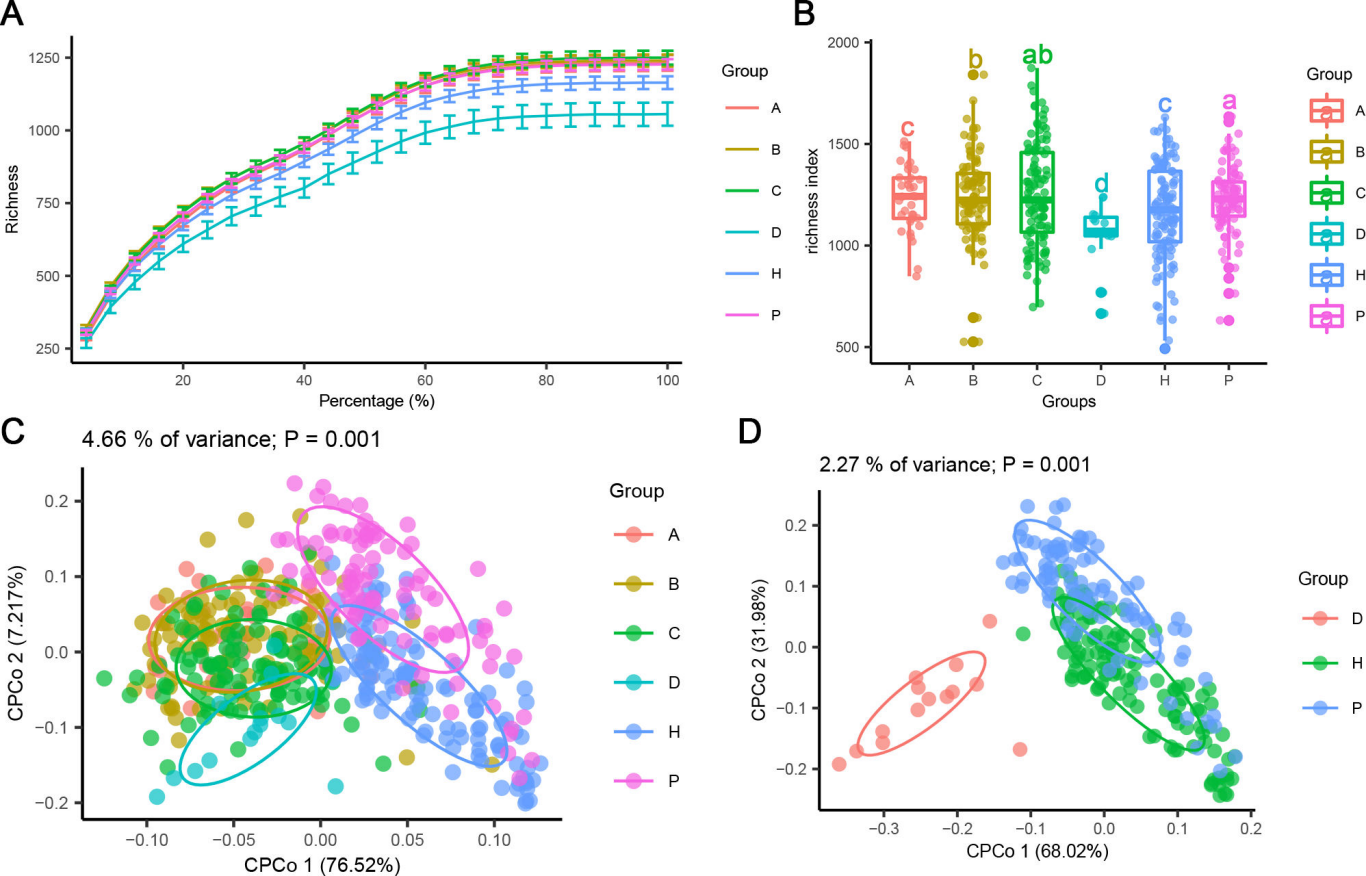
Microbial diversity analysis of the fecal microbiome. (**A**) Rarefaction curve of richness. (**B**) Boxplot of richness index of α-diversity. (**C**) CPCoA plot based on Bray-Curtis matrix of β-diversity among the C1, C2, C3, C4, H, and P groups. (**D**) CPCoA plot based on Bray-Curtis matrix of β-diversity among the C4, H. and P groups. The data are presented as the mean ± standard deviation. Boxplot (**B**) marked with different letters on top indicates statistically significant differences as determined by one-way ANOVA at a significance level of *P* < 0.05. ANOVA, analysis of variance; CPCoA, constrained principal coordinate analysis.

The diversity of microbial communities of fecal samples from patients with different stages of CRC, healthy people, and patients with polyps was analyzed by 16S rRNA sequencing. The rarefaction curve tended to a stable value, indicating that the amount of our sequencing data were reasonable (Fig. 1A). The α-diversity results indicated that the richness index was significantly reduced in patients with CRC stage IV compared with other groups. Compared with the P group, the richness index was significantly reduced in patients with CRC stages I, II, and IV, and the healthy group (Fig. 1B). For β-diversity (Fig. 1C), CPCoA plot based on the Bray-Curtis matrix indicated a significant difference between the patients with CRC and healthy people or patients with polyps (*P* = 0.001; ANOISM, permutations 999). The β-diversity analysis conducted in patients with CRC stage IV, polyp patients, and healthy controls exhibited significant differences between patients with CRC stage IV and the other two groups (*P* = 0.001; ANOISM, permutations 999) (Fig. 1D).

### Comparison of fecal microbial composition

**Fig 2:**
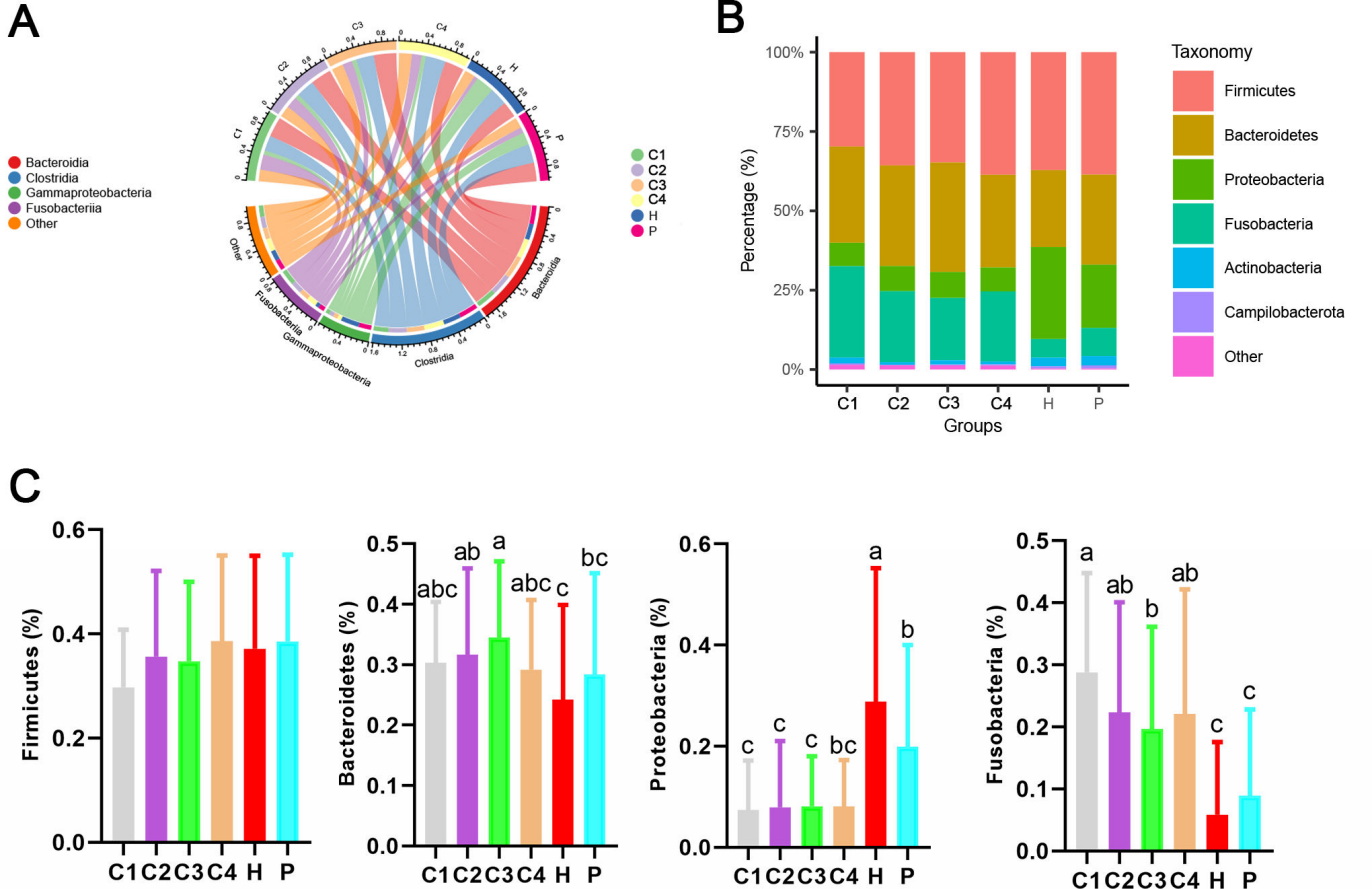
Microbial composition of the fecal microbiome at phylum and class levels. (**A**) Chord diagram at the class level. (**B**) Bar chart of relative abundance for phylum level. (**C**) Boxplot of the four dominant phyla. The data are presented as the mean ± standard deviation. Boxplot (**C**) marked with different letters on top indicates statistically significant differences as determined by one-way ANOVA at a significance level of *P* < 0.05. ANOVA, analysis of variance.

The chord diagram at the class level showed that Bacteroidia, Clostridia, Gammaproteobacteria, and Fusobacteria were the dominant class in the six groups of volunteers (Fig. 2A). At the phylum level, Firmicutes, Proteobacteria, Bacteroidetes, and Fusobacteria were the dominant phylum of the six groups of volunteers (Fig. 2B). From the overall trend, the relative abundance of Firmicutes and Proteobacteria was increased, while Bacteroidetes and Fusobacteria were reduced in the H group compared to the patients with CRC and P groups (Fig. 2B). The relative abundance of Firmicutes was not significantly different among the six groups, but showed an upward trend in patients with CRC (Fig. 2C). Compared with healthy people, the relative abundance of Bacteroidetes was significantly increased in patients with CRC stages II and III. The relative abundance of Bacteroidetes in fecal microorganisms was significantly higher in patients with CRC stage III compared with the P group (Fig. 2C). The relative abundance of Proteobacteria was significantly higher in the H group than in the other groups. The relative abundance of Proteobacteria in group P was significantly higher than in the C1, C2, and C3 groups (Fig. 2C). The relative abundance of Fusobacteria was significantly increased in patients with CRC compared with the H and P groups. Fusobacteria relative abundance was significantly higher in patients with CRC stage I than in CRC stage III (Fig. 2C).

### Diagnosis model for diagnosing different health status

**Fig 3:**
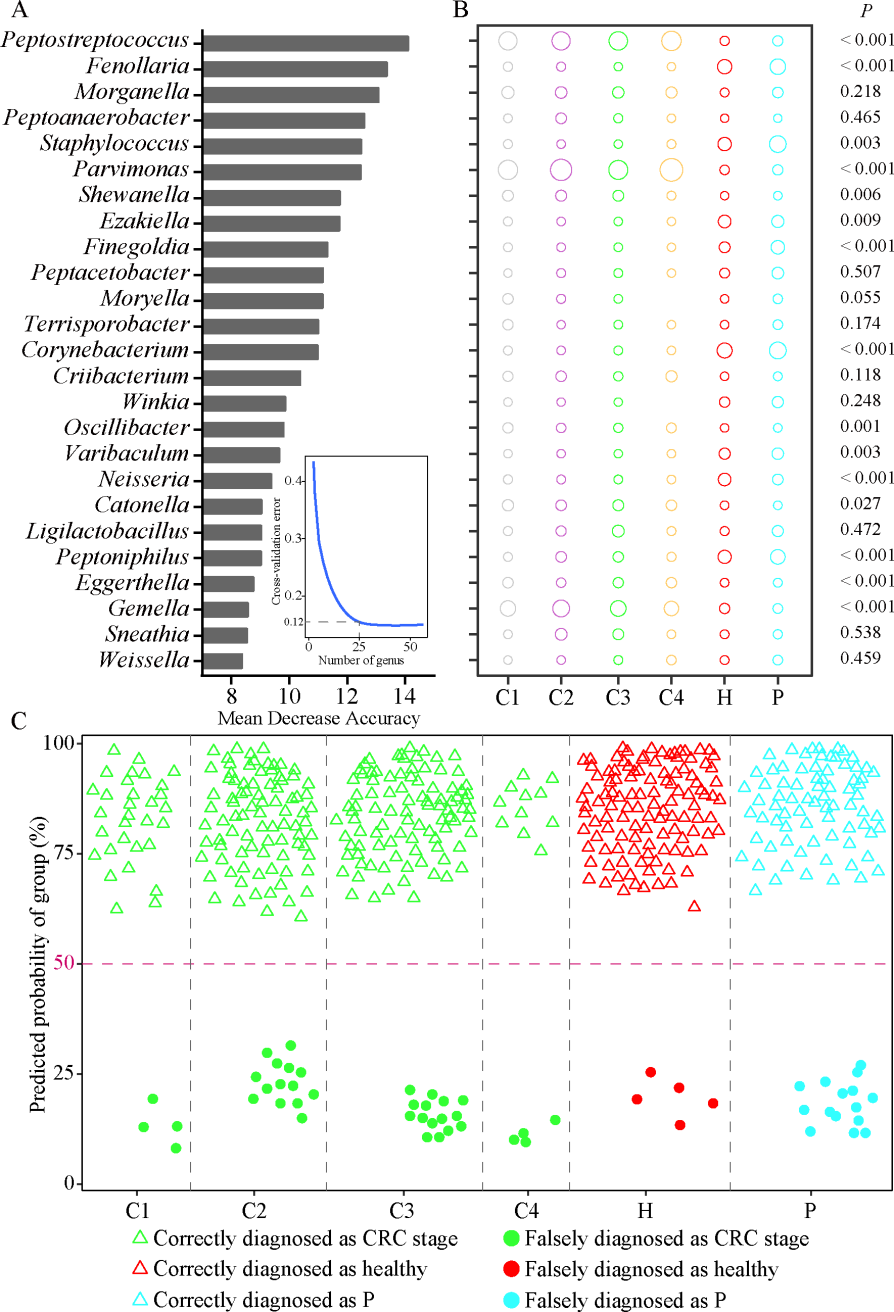
RF model was used to diagnose patients with CRC stages I, II, III, and IV, healthy people, and patients with polyps using differential classification of intestinal microbiota at the genus level. (**A**) The top 25 genera were ranked in descending order according to their relative importance for diagnostic model accuracy using a 10-fold cross-validation method. (**B**) Relative abundance ratios of 25 genera between the six groups. (**C**) Predicted probabilities for each group. CRC, colorectal cancer; RF, random forest.

The gut microbiota was significantly affected by health status (ANOSM, *P* < 0.001) and gender (ANOSM, *P* < 0.001), but not by age (*P* = 0.483) (Table S1). Therefore, stage of CRC and gender were considered the conditional factors. We identified the top 25 genera from 174 training data (Fig. 3A), resulting in a diagnostic accuracy of 87.95% for different stages of CRC, healthy people, and patients with polyps (Table 1). Overall, *Peptostreptococcus*, *Parvimonas*, *Shewanella*, *Oscillibacter*, *Catonella*, *Eggerthella,* and *Gemella* were significantly enriched in patients with CRC compared to healthy cohorts (Fig. 3B). *Fenollaria*, *Staphylococcus*, *Ezakiella*, *Finegoldia*, *Corynebacterium*, *Varibaculum*, *Neisseria,* and *Peptoniphilu*s were significantly enriched in healthy individuals and patients with polyps (Fig. 3B). It was important to note that the diagnostic accuracy was not compromised by low sample size (Fig. 3C). In addition, we identified the top seven different genera diagnosing gender of subjects from 174 training data, including *Prevotellamassilia*, *Paraclostridium*, *Devosia*, *Aeromonas*, *Shigella*, *Faecalimonas*, and *Alloprevotella* (Fig. S1).

**TABLE 1:** Diagnostic accuracy of genera based on 25 CRC discriminations in 473 subjects

Observed	Predicted						Overall accuracy
	C1	C2	C3	C4	H	P	
C1	31	1	1	0	0	2	(416/473) 87.95%
C2	0	87	10	0	0	4	
C3	0	15	95	0	0	0	
C4	0	4	0	11	0	0	
H	0	0	0	0	115	5	
P	3	1	0	0	11	77	

### Key differential genera from diagnosis model

**Fig 4:**
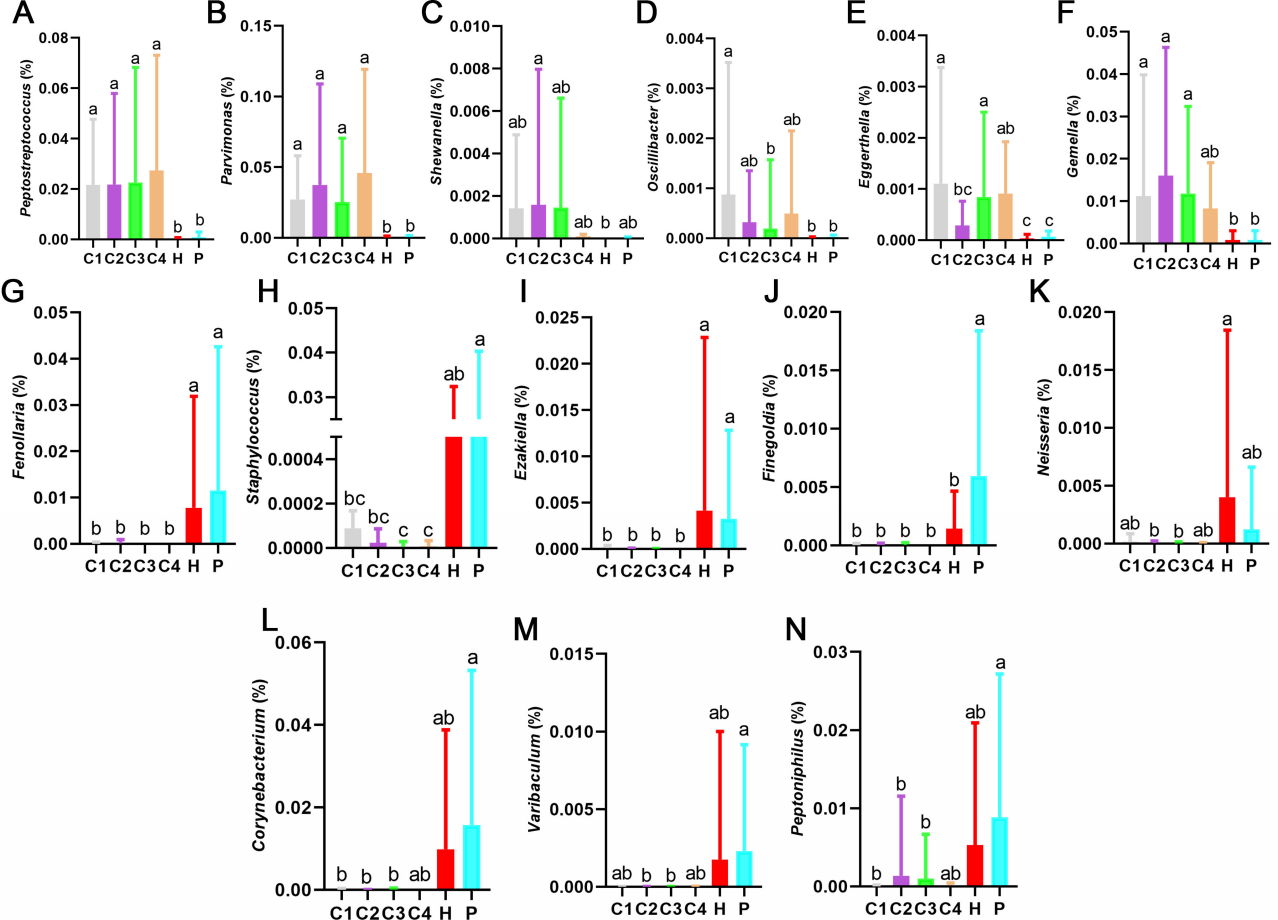
Microbial composition of 14 key differential genera from diagnosis model. (**A**) Boxplot of the relative abundance of *Peptostreptococcus*. (**B**) Boxplot of the relative abundance of *Parvimonas*. (**C**) Boxplot of the relative abundance of *Shewanella*. (**D**) Boxplot of the relative abundance of *Oscillibacter*. (**E**) Boxplot of the relative abundance of *Eggerthella*. (**F**) Boxplot of the relative abundance of *Gemella*. (**G**) Boxplot of the relative abundance of *Fenollaria*. (**H**) Boxplot of the relative abundance of *Staphylococcus*. (**I**) Boxplot of the relative abundance of *Ezakiella*. (**J**) Boxplot of the relative abundance of *Finegoldia*. (**K**) Boxplot of the relative abundance of *Neisseria*. (**L**) Boxplot of the relative abundance of *Corynebacterium*. (**M**) Boxplot of the relative abundance of *Varibaculum*. (**N**) Boxplot of the relative abundance of *Peptoniphilus*. The data are presented as the mean ± standard deviation. Boxplot marked with different letters on top indicates statistically significant differences as determined by one-way ANOVA at a significance level of *P* < 0.05. ANOVA, analysis of variance.

We performed boxplot analysis for the key differential genera (Fig. 4). Six differential genera were significantly increased in patients with CRC (Fig. 4A through F). The relative abundance of *Peptostreptococcus* and *Parvimonas* was significantly higher in patients with CRC than in healthy individuals and patients with polyps (Fig. 4A and B). The relative abundance of *Shewanella* in the C2 group was significantly higher than the H group (Fig. 4C). *Oscillibacter* in the C1 group showed a marked increase compared with the H and P groups (Fig. 4D). The relative abundance of *Eggerthella* in patients with CRC stages I and IV was remarkably higher than that of healthy people and patients with polyps (Fig. 4E). The relative abundance of *Gemella* in the C1, C2, and C3 groups was significantly higher than in the H and P groups (Fig. 4F). The relative abundance of *Peptostreptococcus*, *Parvimonas*, *Shewanella*, *Oscillibacter*, *Eggerthella,* and *Gemella* in the C4 group had a 122.1-, 143.6-, 64.3-, 49.7-, 25.4-, and 10.2-fold increase compared with the H group, respectively.

Five differential genera were significantly decreased in patients with CRC (Fig. 4G through N). *Fenollaria and Ezakiella* in the C1, C2, C3, and C4 groups showed a marked decrease compared with the H and P groups (Fig. 4G and I). The relative abundance of *Staphylococcus* in the C3 and C4 groups was significantly lower than in the H and P groups (Fig. 4H). The relative abundance of *Finegoldia* was significantly higher in the P group than in the other groups (Fig. 4J). *Neisseria* had a significant upward trend in the healthy group compared to the C2 and C3 groups (Fig. 4K). The relative abundance of *Fenollaria*, *Staphylococcus*, *Ezakiella*, *Finegoldia*, and *Neisseria* in the H group had a 5,677.4-, 1,085.2-, 7,969.8-, 2,392.6-, and 166.6-fold increase compared to the C4 group, respectively. However, the relative abundance of *Corynebacterium*, *Varibaculum,* and *Peptoniphilu*s did not change significantly between the healthy group and the CRC groups (Fig. 4L through N), suggesting that *Corynebacterium*, *Varibaculum,* and *Peptoniphilu*s could not be used as biomarkers.

### Co-occurrence network analysis at the genus level of fecal microbes from six groups of volunteers

**Fig 5:**
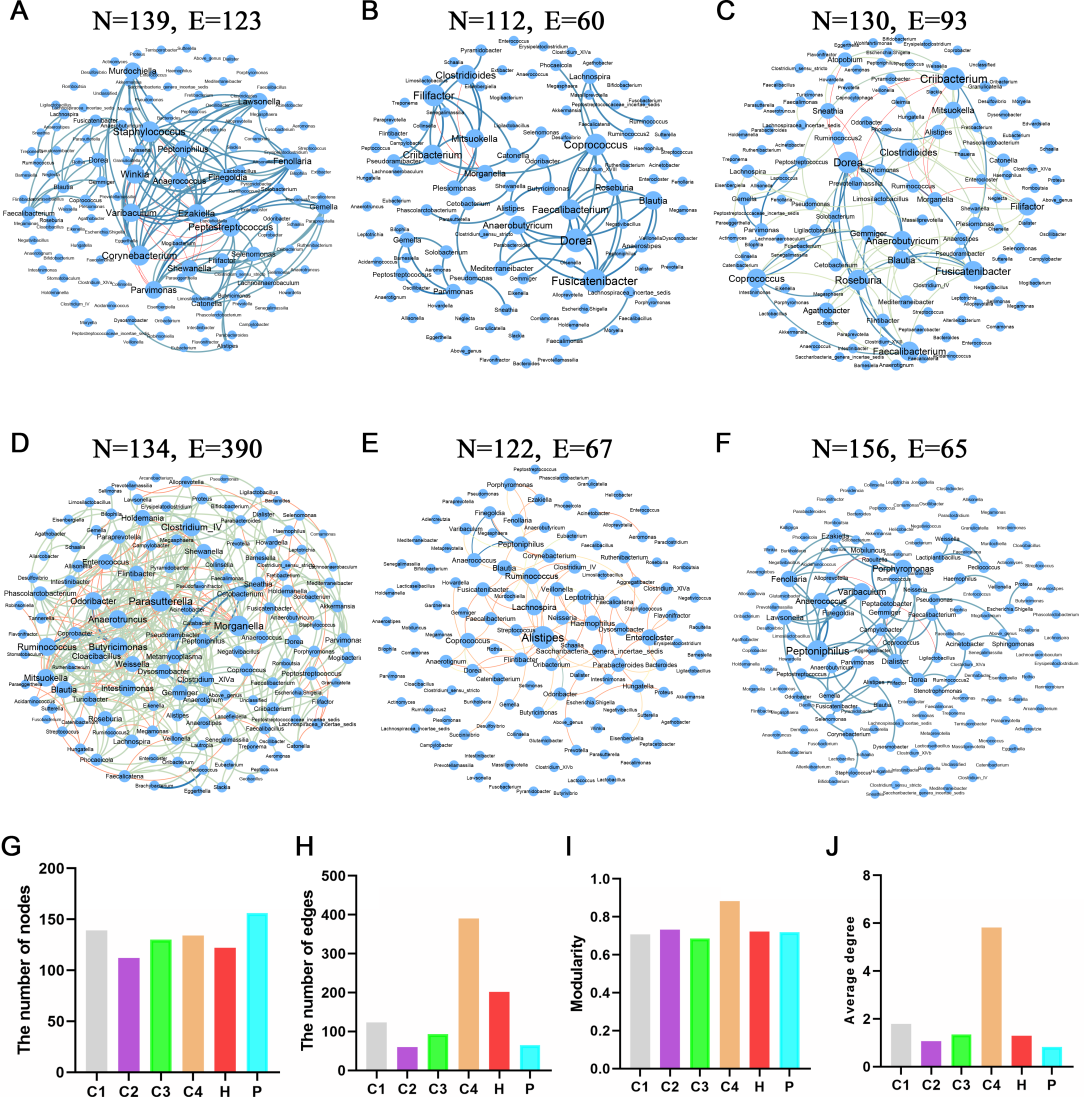
Microbial co-occurrence networks of fecal microbial genera. (**A–F**) Microbial co-occurrence networks of fecal microbial genera in patients with CRC stages I, II, III, and IV, healthy people, and patients with polyps. (**G**) The number of nodes in the fecal microbial co-occurrence networks of six groups. (**H**) The number of edges in the fecal microbial co-occurrence networks of six groups. (**I**) The modularity in the fecal microbial co-occurrence networks of six groups. (**J**) The average degree in the fecal microbial co-occurrence networks of six groups. Nodes represent the microbial genera. Blue edges indicate significant positive interactions, and red edges indicate significant negative interactions.

We performed the co-occurrence network analysis of fecal bacterial genera from six groups. The fecal microbial co-occurrence networks of patients with CRC I (139 nodes and 123 edges), II (112 nodes and 60 edges), III (130 nodes and 93 edges), and IV (134 nodes and 390 edges) were presented in Fig. 5A through D. The fecal microbial co-occurrence network of patients with polyps had 156 nodes and 65 edges (Fig. 5F). However, the nodes and edges in the fecal microbial co-occurrence network of healthy people (122 nodes and 202 edges) were less than CRC I, III, and IV (Fig. 5E). The stability of the co-occurrence network is positively correlated with various metrics of co-occurrence network complexity, such as the number of nodes and edges, modularity, and average degree (38, 39). The modularity of fecal microbial co-occurrence networks from patients with CRC I (0.707), II (0.732), III (0.685), IV (0.883), H group (0.723), and P group (0.720) was presented in Fig. 5I. The average degree of fecal microbial co-occurrence networks from patients with CRC I (1.783), II (1.071), III (1.348), IV (5.821), H group (1.295), and P group (0.833) was presented in Fig. 5J. Notably, the C4 group had the highest number of edges, modularity, and average degree, suggesting that the fecal microbial co-occurrence network of CRC IV was the most complex and stable (Fig. 5G through J).

### Correlation analysis of blood biochemical indicators and microbiota

**Fig 6:**
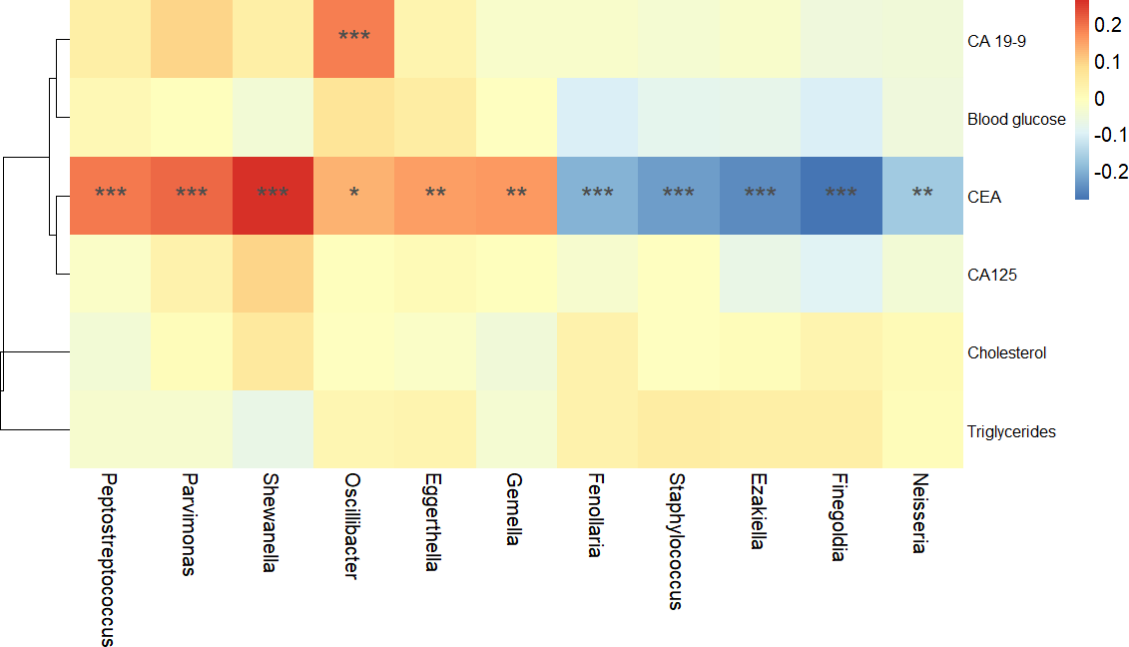
Correlation heatmap analysis of blood biochemical indicators and differential genera. Red represents positive correlation, and blue represents negative correlation. *P < 0.05, **P < 0.01, ***P < 0.001. CA 19-9, carbohydrate antigen 19-9; CEA, carcinoembryonic antigen; CA 125, carbohydrate antigen 125.

In order to explore the relationship between blood biochemical indicators and key differential genera, R software was used to calculate the Spearman correlation coefficient and conduct a correlation heatmap (Fig. 6). Based on the diagnosis model and Fig. 4, 11 differential genera were found to be significantly changed in the H group and the CRC groups, so we selected these 11 differential genera for correlation analysis with blood biochemical indicators. CEA was significantly positively correlated with six differential genera including *Peptostreptococcus*, *Parvimonas*, *Shewanella*, *Oscillibacter*, *Eggerthella*, and *Gemella* (Fig. 6). In addition, there were significant negative correlations between CEA and *Fenollaria*, *Staphylococcus*, *Ezakiella*, *Finegoldia*, and *Neisseria* (Fig. 6). CA 19-9 was significantly positively correlated with *Oscillibacter* (Fig. 6).

## DISCUSSION

CRC is the second leading cause of cancer mortality worldwide, and its incidence is increasing in younger age groups (40). An important issue with CRC is racial disparity, with higher rates in Europe and North America. This difference may be related to differences in the gut microbiome (41). By comparing the whole-genome sequencing data of gut bacteria from six countries, including Japan, China, the United States of America, Germany, and France, there are differences in dominant genera at the genus level among different countries. In China, studies on the role of gut microbiota in CRC showed that *Fusobacterium nucleatum* and *Parvimonas micra* were significantly enriched in patients with CRC (42). An abnormal increase in *Porphyromonas* was associated with CRC in a Japanese study (43). Region is an important factor leading to the differences in CRC-related gut microbiota (44).

To date, a clear link between *Helicobacter pylori* and gastric cancer has been found in the previous study (45). Although no direct causal relationship has been found between a specific fecal microbe and CRC, the potential link between fecal microbial imbalance and CRC remains a focus of research. Exploring how the imbalance of fecal microbiota affects the development of CRC and how these changes are linked to blood indicators was important for revealing the pathogenesis of CRC and developing preventive strategies. This study aimed to identify the key fecal microbes and blood indicators that will help understand the progression of CRC and provide scientific evidence for the prevention and treatment of CRC. Through an in-depth understanding of the interaction between fecal microbiota and CRC, new diagnostic tools and treatments have been developed to reduce CRC morbidity and mortality.

The microbial composition in feces is not only a mapping of gut bacterial species, but also reveals the functions of gut bacteria in the tumor microenvironment (46). By analyzing fecal samples, we can gain insights into the role of fecal bacteria in CRC development. Our study found that patients with CRC stage IV had significantly lower α-diversity than other groups (Fig. 1B). Similar to our results, patients with CRC have lower fecal microbiome α-diversity (47). The reduction of critical microbial species may lead to an imbalance in fecal homeostasis and a reduction in microbial diversity (48). In addition, the reduced microbial diversity in the feces of patients with polyps may be associated with a reduced immune response (19). The stability and resilience of fecal microbiota are key factors in maintaining intestinal health. High microbial diversity is often associated with increased functional redundancy, stabilizing the function of the microbiota in response to perturbations (49). When this microecological balance is disturbed, it can trigger a variety of diseases, including inflammatory bowel disease and CRC (50). In our study, β-diversity analysis revealed significant differences in fecal microbiota structure in patients with CRC and those with polyps compared to healthy people (Fig. 1C and D). The changes in α-diversity and β-diversity reflected the imbalance of fecal microbiota.

Human gut microbiota is a diverse ecological network that plays a key role in maintaining intestinal health, participating in key physiological processes such as digestion, metabolism, and immune regulation, and maintaining the barrier function of the gut (51). At the phylum level, Firmicutes, Bacteroidetes, Proteobacteria, and Fusobacteria are the most abundant bacterial communities, which together constitute the core of the intestinal microbial community (52). Our findings indicated that the H group had higher relative abundance of Firmicutes and Proteobacteria and lower relative abundance of Bacteroidetes and Fusobacteria compared to patients with CRC and the *P* group (Fig. 2B). The reduction of Firmicutes may affect the host immune metabolism, thereby affecting the alleviation of CRC (53). Specific species of the Bacteroidetes (*Bacteroidetes dorei*, *Bacteroidetes fragilis*, and *Bacteroidetes thetaiotaomicron*) are increased in obese patients with CRC, which can accelerate the development of CRC (54). Some intestinal microorganisms belonging to the Proteobacteria can promote CRC development (55). Another study also mentions that Proteobacteria show a decreasing trend from CRC stages I to IV (56), suggesting that the relative abundance of Proteobacteria may be reduced in patients with CRC. Fusobacteria is closely related to the occurrence and development of CRC, and the relative abundance of Fusobacteria in patients with CRC is significantly higher than in healthy people (57).

Previous studies have shown that fecal microbial co-occurrence networks are more complex in patients with CRC than in healthy people (58). The microbial co-occurrence networks of patients with CRC I, III, and IV were more complex than that of the healthy people, and the co-occurrence network of CRC IV was the most complex and stable (Fig. 5), which was consistent with the previous studies. Our microbial co-occurrence networks showed that *Blautia*, *Ruminococcus*, *Dorea*, *Parvimonas*, *Peptostreptococcus*, *Selenomonas*, and *Porphyromonas* were highly abundant in the patients with CRC (Fig. 5A through F). These harmful bacteria are associated with the clinical characteristics of patients with CRC (59). *Blautia* is significantly enriched in the mice model of CRC (60). *Ruminococcus* and *Dorea* are significantly enriched in colon tissues of patients with CRC (61). *Parvimonas* is one of the gut bacteria associated with CRC (62). *Peptostreptococcus* can promote spontaneous CRC in mice by inducing cell proliferation, inhibiting cell apoptosis, and damaging the intestinal mucosal barrier (63). *Selenomonas* is present in metastatic tumors, suggesting that *Selenomonas* may be involved in CRC metastasis (64). There is a causal relationship between *Porphyromonas* overgrowth and colorectal tumorigenesis (43).

By RF, 25 genera screened for diagnosing different stages of patients with CRC, healthy people, and patients with polyps (Table 1; Fig. 3) showed high diagnostic accuracy (87.95%). Furthermore, 11 differential genera significantly changed between the H group and the CRC groups, providing a reliable quantitative method for the diagnosis of human health conditions. The CRC-closely associated bacterial genera (*Peptostreptococcus*, *Parvimonas*, *Shewanella*, *Oscillibacter*, *Eggerthella*, and *Gemella*) were significantly enriched in the fecal microbiota of patients with CRC (Fig. 4A through F), suggesting they may serve as potential CRC markers. *Peptostreptococcus* promotes spontaneous CRC by damaging the intestinal barrier, inhibiting cell apoptosis, and inducing cell proliferation (63). *Peptostreptococcus* is a common high-risk pathogen of CRC worldwide and an important variable for CRC risk prediction models in various regions (44). *Parvimonas* is one of the gut bacteria associated with CRC (62). *Shewanella* has been shown to be associated with the development of CRC (65). *Oscillibacter* is shown to be increased in relative abundance in mice with CRC (66). *Eggerthella* has been reported as a CRC marker and is abundant in patients with CRC (67). In patients with CRC, the number of *Gemella* is significantly increased, which is closely related to the development and progression of CRC (68).

The bacterial genera including *Fenollaria*, *Staphylococcus*, *Ezakiella*, *Finegoldia,* and *Neisseria* were significantly decreased in the fecal microbiota of patients with CRC (Fig. 4G through K), implying that the reduction of these beneficial bacterial genera may contribute to the development of CRC. *Fenollaria* can exist in the human body as part of the normal intestinal microbiota and may play a role in maintaining the balance and health of intestinal microbiota (69). In 2018, the genus *Fenollaria* and its type species *Fenollaria massiliensis* were officially and effectively published in the international journals (70). *Staphylococcus* and its secretions may have potential therapeutic effects on cancer (71). *Ezakiella* and *Finegoldia* play an important role in maintaining the health and stability of bacterial flora (72, 73). Previous studies find that *Neisseria* are significantly decreased in the patients with CRC (74).

CEA, as a tumor biomarker and a prognostic biomarker, is widely used in the diagnosis and treatment monitoring of CRC. The increased CEA levels may predict disease deterioration or recurrence, making it an important biomarker to track the disease progression of CRC (75). Postoperative monitoring of CEA changes is equally important for detecting disease recurrence and metastasis, and necessary measures can be taken earlier to deal with possible disease deterioration (76). CA 19-9 is a biomarker for the clinical management of patients with gastrointestinal cancer. The level of CA 19-9 may be significantly increased in pancreatic cancer, hepatobiliary cancer, gastric cancer, and CRC (77). In Europe, CEA is widely used for CRC screening, diagnosis, and monitoring of recurrence (78). Asian studies also support the application of CEA as a CRC marker, especially in the early detection and prognostic evaluation of CRC (79, 80). In our results, CEA was positively correlated with *Peptostreptococcus*, *Parvimonas*, *Shewanella*, *Oscillibacter*, *Eggerthella*, and *Gemella*, which were six harmful bacteria linked to the occurrence and development of CRC. CEA was also significantly negatively correlated with *Fenollaria*, *Staphylococcus*, *Ezakiella*, *Finegoldia*, and *Neisseria* (Fig. 6), five beneficial bacteria that were related to cancer treatment. CA19-9 was positively markedly correlated with *Oscillibacter* (Fig. 6).

### Conclusion

In conclusion, our study highlighted the role of microorganisms in the human fecal microbiome that were strongly associated with CRC. Fecal microbiota of patients with CRC was disturbed, as evidenced by significantly reduced α-diversity in patients with CRC stage IV and markedly different β-diversity between the patients with CRC and healthy people or patients with polyps. The RF model and differential genera analysis were used to identify eleven biomarkers that significantly changed in different stages of patients with CRC, healthy people, and patients with polyps. The identified microbial biomarkers highlighted the potential of microbiota-based models for non-invasive CRC diagnosis in correlation with blood biochemical biomarkers. These results suggested that CEA, CA19-9, and these key fecal microbes including *Peptostreptococcus*, *Parvimonas*, *Shewanella*, *Oscillibacter*, *Eggerthella*, *Gemella*, *Fenollaria*, *Staphylococcus*, *Ezakiella*, *Finegoldia,* and *Neisseria* may be co-biomarkers for the disease occurrence, development, and non-invasive diagnosis of CRC.

## Data Availability

The raw sequence data used in this analysis have been uploaded to the Genome Sequence Archive in the BIG Data Center, Chinese Academy of Sciences under accession code CRA012037 at http://bigd.big.ac.cn/gsa.

## SUPPLEMENTAL MATERIAL

Supplemental material msystems.00043-25-s0001.docx Table S1 and Figure S1.

ASM does not own the copyrights to Supplemental Material that may be linked to, or accessed through, an article. The authors have granted ASM a non-exclusive, world-wide license to publish the Supplemental Material files. Please contact the corresponding author directly for reuse.
