# Supplementary material for: Fecal bacterial biomarkers and blood biochemical indicators as potential key factors in the development of colorectal cancer
Source: mSystems. 2025 Feb 27;10(3):e00043-25. doi: 10.1128/msystems.00043-25 (PMC11915818; doi:10.1128/msystems.00043-25)
Supplement: Supplemental material — Table S1 and Figure S1. [file msystems.00043-25-s0001.docx]

Fecal bacterial biomarkers and blood biochemical indicators as potential key factors in the development of colorectal cancer

Ping Cai^1,2^, Qingzhen Yang^2,3^, Jiaqi Lu^4^, Xiaoyu Dai^1*^, Jinbo Xiong^5*^

^1^ Ningbo No.2 Hospital, Ningbo, China;

^2^ The Key Laboratory of Biomedical Information Engineering of Ministry of Education, School of Life Science and Technology, Xi’an Jiaotong University, Xi’an, China;

^3^ Bioinspired Engineering and Biomechanics Center (BEBC), Xi’an Jiaotong University, Xi’an, China;

^4^ Zhejiang KinGene Bio-technology Co., Ltd, Ningbo, China;

^5^ Institute of One Health, School of Marine Sciences, Ningbo University, Ningbo 315211, China.

Running Head: Colorectal Cancer Biomarkers.

The word count of the manuscript abstract is 248.

***Corresponding authors**

Xiaoyu Dai, E-mail: daixiaoyu1968@163.com

Address correspondence to Jinbo Xiong, E-mail: [xiongjinbo@nbu.edu.cn](mailto:xiongjinbo@nbu.edu.cn)

**Table S1.** Different stages of colorectal cancer, age and gender of volunteers were studied. The impact was measured using analysis of similarity (ANOSIM, R^2^ value). The R^2^ values represent the proportion of the community variation constrained by each variable and their interaction. For each analysis we performed 999 permutations to obtain the *P* value.

|  | R^2^ | *P* |
| --- | --- | --- |
| Stage | 0.022 | < 0.001 |
| Age | 0.002 | 0.483 |
| Gender | 0.074 | < 0.001 |
| Stage: age | 0.010 | 0.360 |
| Stage: gender | 0.025 | < 0.001 |
| Age: gender | 0.002 | 0.261 |


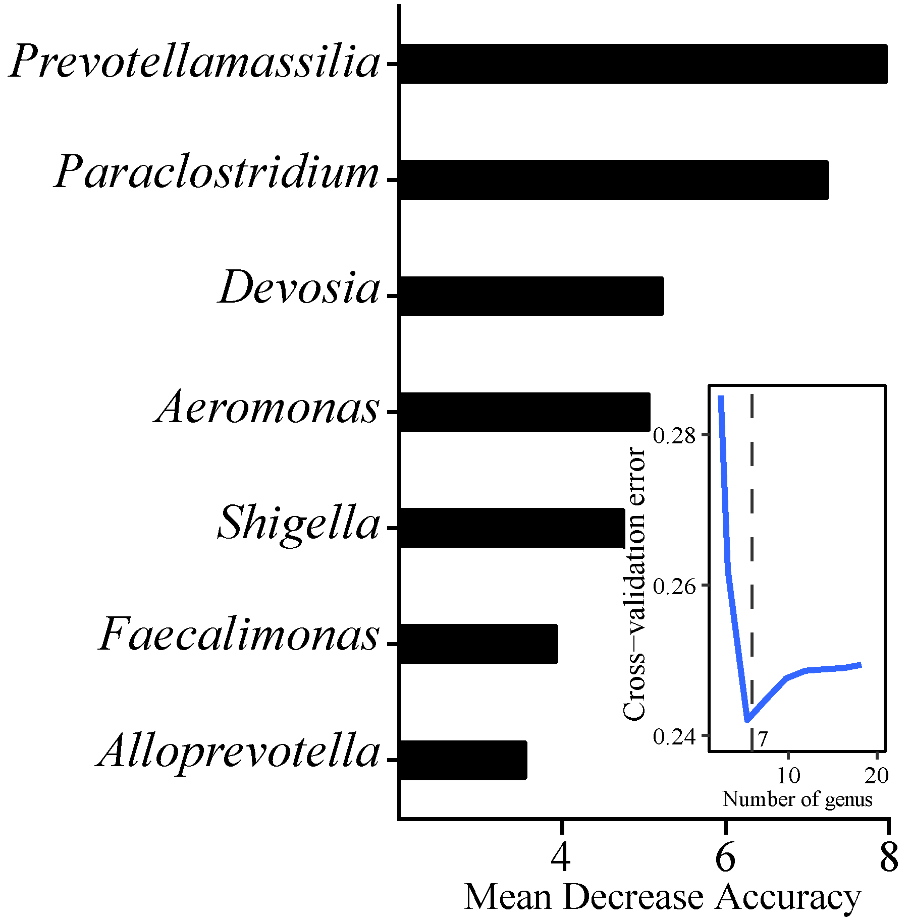


**Figure S1.** Random Forest model was used to diagnose gender of subjects using differential classification of intestinal microbiota at the genus level. The top seven genera were ranked in descending order according to their relative importance for diagnostic model accuracy using a 10-fold cross-validation method.
